# Do precipitation anomalies influence short-term mobility in sub-saharan Africa? An observational study from 23 countries

**DOI:** 10.1186/s12889-023-15264-z

**Published:** 2023-02-22

**Authors:** Adrienne Epstein, Orlando O. Harris, Tarik Benmarhnia, Carol S. Camlin, Sheri D. Weiser

**Affiliations:** 1 Department of Vector Biology, Liverpool School of Tropical Medicine, Pembroke Place, L3 5QA Liverpool, UK; 2 Department of Epidemiology and Biostatistics, University of California, San Francisco, USA; 3 Department of Medicine, Center for AIDS Prevention Studies, University of California, San Francisco, USA; 4 Herbert Wertheim School of Public Health and Human Longevity Science & Scripps Institution of Oceanography, University of California, San Diego, USA; 5 Department of Obstetrics, Gynecology & Reproductive Sciences, University of California, San Francisco, USA; 6 Division of HIV, Infectious Diseases and Global Medicine, University of California, San Francisco, USA

**Keywords:** Droughts, Rain, Demography, Africa South of the Sahara, Migration

## Abstract

**Background:**

Precipitation anomalies are associated with a number of poor health outcomes. One potential consequence of precipitation extremes is human geographic mobility. We evaluated the associations between precipitation anomalies (droughts and heavy rains) and short-term mobility in 23 sub-Saharan African countries by linking satellite data on precipitation to cross-sectional representative surveys.

**Methods:**

Using data from 23 Demographic and Health Surveys from 2011 to 2017, we estimated the associations between deviations in long-term rainfall trends and short-term mobility among 294,539 women and 136,415 men over 15 years of age. We fit multivariable logistic regression models to assess potential non-linear relationships between rainfall deviations and short-term mobility, adjusting for survey month and socio-demographic covariates, and stratified by participant gender. Furthermore, we assessed whether these associations differed by marital status.

**Results:**

Rainfall deviations were associated with short-term mobility among women, but not men. The relationship between rainfall deviations and mobility among women was U-shaped, such that women had increased marginal probabilities of mobility in instances of both lower and heavier precipitation. Differences between married and unmarried women were also revealed: among married women, we found positive associations between both rainfall deviation extremes (drought and heavy rains) and mobility; however, among unmarried women, there was only a positive association for heavy rains.

**Conclusion:**

Precipitation anomalies were associated with short-term mobility among women, which may be in turn associated with poor health outcomes. More research with longitudinal data is needed to elaborate the associations between weather shocks, mobility, and downstream health impacts.

**Supplementary Information:**

The online version contains supplementary material available at 10.1186/s12889-023-15264-z.

## Background

Precipitation anomalies are increasing in frequency and intensity around the globe [1]. These events are associated with poor health consequences including malnutrition, infectious diseases, non-communicable diseases and depression [2, 3] One often cited consequence of precipitation anomalies that is lacking empirical evidence is forced human geographic mobility. That mobility is composed of both migration, or changes of residence over defined geographic boundaries (i.e. international migration or moves across nation states, and internal migrations or within-country moves), and other more complex mobility flows, including temporary and localized movements to and from households of origin.

Extreme precipitation may lead to mobility through loss of arable land, damages to housing and infrastructure, reduced income, water insecurity, and inadequate food supply [4]. Case studies have linked drought to mobility in several settings including Mali, where drought was associated with short-term migration by women and children [5], Mexico, where drought was associated with urban-rural migration [6], Ethiopia, where drought was associated with increased men’s labor migration and declines in women’s marriage-related migration [7], and Haiti, where drought, poverty, and deforestation were associated with migration to the Dominican Republic [8]. While there is less empiric evidence linking heavy rain to mobility, there is some evidence that these events have modest impacts on human mobility in Bangladesh [9]. In other settings, floods and heavy rains were shown to have no impact on mobility [10]. Measuring the association between precipitation extremes and mobility remains challenging due to limited data on migration flows both within and in-and-out of regions and countries [11]. Therefore, to our knowledge, no studies have pooled data across countries to explore the link between precipitation extremes and mobility, and few have simultaneously studied both extremes (e.g., drought and heavy rains) [10]. Understanding this link is vital in sub-Saharan Africa, where 363 million people were impacted by drought from 1980 to 2014 and increases in short, intense rain storms have been documented [12, 13].

Environmental mobility – mobility and migration in response to sudden or long-term changes in their environment – can lead to vulnerabilities to poor health. Unlike voluntary migration and mobility, which has demonstrated benefits to household livelihoods and child health [14, 15], environmental mobility is a form of forced mobility. Poor health outcomes associated with environmental mobility include worse mental health, non-communicable disease, and infectious disease, deemed a “triple burden” [16, 17]. Women are particularly vulnerable to the health impacts of mobility, as they often face barriers to reproductive care [18] and adequate sanitation [19], are more likely to engage in higher risk sexual behavior and therefore risk exposure to HIV [20, 21], and face elevated risks of violence and sexual abuse [19, 22]. Furthermore, mobility in response to environmental stress may be influenced by marital status, as married couples may have to make decisions regarding mobility jointly.

We use nationally-representative data from 23 sub-Saharan African countries to explore the relationship between precipitation anomalies and short-term mobility. Given the gendered differences in the drivers, patterns, and consequences of mobility, we evaluate whether these relationships differ between men and women. We also assess whether this associations differ by marital status.

## Methods

*Data source.* This analysis used data from Demographic and Health Surveys (DHS) in sub-Saharan Africa. DHS are nationally-representative surveys. All women in selected households are invited to participate, while men are recruited in a subsample of randomly selected households. We included surveys that occurred during or after 2011 through 2017 (due to the availability of precipitation data) and that geocoded enumeration areas (EA). This analysis included 23 sub-Saharan African countries (Table 1). We included men and women with full covariate and outcome data.

**Table 1:** Survey and sample sizes included in analysis

Country	Survey year	Sample size of women	Sample size of men
Angola	2015-16	14,278	5,675
Burundi	2016-17	17,170	7,533
Cameroon	2011	15,153	7,100
Chad	2014-15	17,291	5,155
Côte d’Ivoire	2011-12	9,766	4,963
Democratic Republic of Congo	2013-14	17,314	7,934
Gabon	2012	8,278	5,543
Ghana	2014	9,279	4,316
Guinea	2012	9,122	3,770
Kenya	2014	14,623	12,702
Lesotho	2014	6,609	2,925
Malawi	2015-16	10,411	4,395
Mali	2012–2013	13,704	2,875
Mozambique	2011	24,522	7,461
Namibia	2013	9,895	4,407
Rwanda	2014-15	13,483	6,203
Sierra Leone	2013	16,616	7,250
Tanzania	2015-16	13,259	3,511
Togo	2013-14	9,449	4,460
Uganda	2016	18,103	5,218
Zambia	2013-14	16,287	14,638
Zimbabwe	2015	9,927	8,381

*Measures.* To quantify precipitation anomalies, we used Climate Hazards Group InfraRed Precipitation Station data, which combines both satellite and station data to interpolate rainfall globally from 1981 to present [23]. For each unique survey date/EA combination, we summed the quantity of rainfall that occurred over the previous 12 months. We then ranked this quantity relative to the quantity of annual rainfall at the EA level over the 29 previous years and converted this ranking to an empirical percentile. For example, a value of 0.5 represents the median level of annual rainfall over the past 30 years. We used this continuous percentile deviation measure as the exposure variable; lower numbers (approaching 0) therefore represent drier times, and higher numbers (approaching 1) represent heavier rainfall in the year prior to the survey relative to the previous 29 years. This classification of rainfall deviations as a relative percentile is standard [24–27].

The outcome was short-term mobility, defined as being away from the participant’s place of residence for longer than one month over the year prior to the survey. Respondents were asked “In the last 12 months, have you been away from your home community for more than a month at a time?” We considered this variable dichotomous [28].

We adjusted for socio-demographic covariates that may impact short-term mobility. Covariates include age (continuous), wealth quintile (categorical; assessed using an asset index [29]), household size (categorical; 1–2, 3–4, 5–7, and 8+), education level (categorical; none, primary secondary, and higher) and binary indicators for urban (versus rural), currently married, and literate. We also included an indicator variable for survey month to adjust for seasonal changes in short-term mobility.

*Statistical analysis*. We fit multivariable logistic regression models to assess the relationship between rainfall anomalies and short-term mobility. To assess non-linearities, we modeled rainfall deviations using restricted cubic splines, with the number of knots determined using Akaike’s information criterion. Models were fit separately for men and women. All models included country-level fixed effects and standard errors were clustered at the EA level. Because we included country fixed effects, our models are “within” estimators, comparing survey participants with different rainfall exposures within each country. To calculate relative risks, we computed marginal predicted probabilities of short-term mobility among participants living at lower rainfall relative to the prior 29 years (percentile of 0.15) and at higher rainfall relativive to the prior 29 years (percentile of 0.85) and compared these to participants living in the median level of rainfall (percentile of 0.5). We then compared marginal predicted probabilities at the extremes to probabilities at the median. To assess effect modification by marital status, we generated interaction terms between rainfall percentile deviations and a binary variable representing marital status. We considered effect modification present if the spline-marital status interaction terms had p-values that were jointly < 0.05. Analyses were carried out in R-Cran version 3.4 and Stata version 14.2.

*Patient and public involvement.* Patients and/or the public were not involved in the design, or conduct, or reporting, or dissemination plans of this research.

## Results

The sample included 294,539 women and 136,415 men. Table 2 shows the characteristics of men and women included in the sample. Approximately two-thirds of participants lived in rural areas and the mean age of participants was 30.7 for men (SD 11.8) and 28.5 for women (SD 9.6). The prevalence of literacy was higher in men (66.5%) than women (53.2%). A total of 12.4% of women and 16.0% of men migrated for at least one month in the year prior to the survey. The mean rainfall deviation for women participants was 0.45 (SD 0.29) and for men participants 0.45 (SD 0.28).

**Table 2:** Descriptive statistics of participants included in analyses

Variable	Women (n = 294,539)	Men (n = 136,415)
Rural, % (n)	62.3 (183,561)	60.8 (82,886)
Age, mean (SD)	28.5 (9.6)	30.7 (11.8)
Household size, % (n)		
1–2	7.0 (20,623)	13.2 (18,013)
3–4	24.2 (71,340)	22.6 (30,313)
5–7	40.1 (117,999)	37.3 (50,898)
8+	28.7 (84,577)	27.9 (36,686)
Wealth quintile, % (n)		
Poorest	19.0 (56,049)	18.3 (24,935)
Poorer	18.5 (54,372)	18.8 (25,594)
Middle	19.0 (56,077)	19.5 (26,567)
Richer	20.6 (60,512)	20.8 (28, 321)
Richest	22.9 (67,529)	22.7 (30,998)
Education level, % (n)		
None	26.5 (78,155)	15.3 (20,858)
Primary	37.9 (111,757)	35.9 (41,942)
Secondary	31.6 (93,204)	41.5 (56,604)
Higher	3.9 (11,423)	7.3 (10,011)
Literate, % (n)	53.2 (156,631)	66.5 (90,762)
Married, % (n)	48.5 (142,797)	44.8 (61,111)
Short-term mobility in past year, % (n)	12.4 (36,596)	16.0 (21,835)
Rainfall deviation percentile, mean (SD)	0.45 (0.29)	0.45 (0.28)

**Fig. 1:**
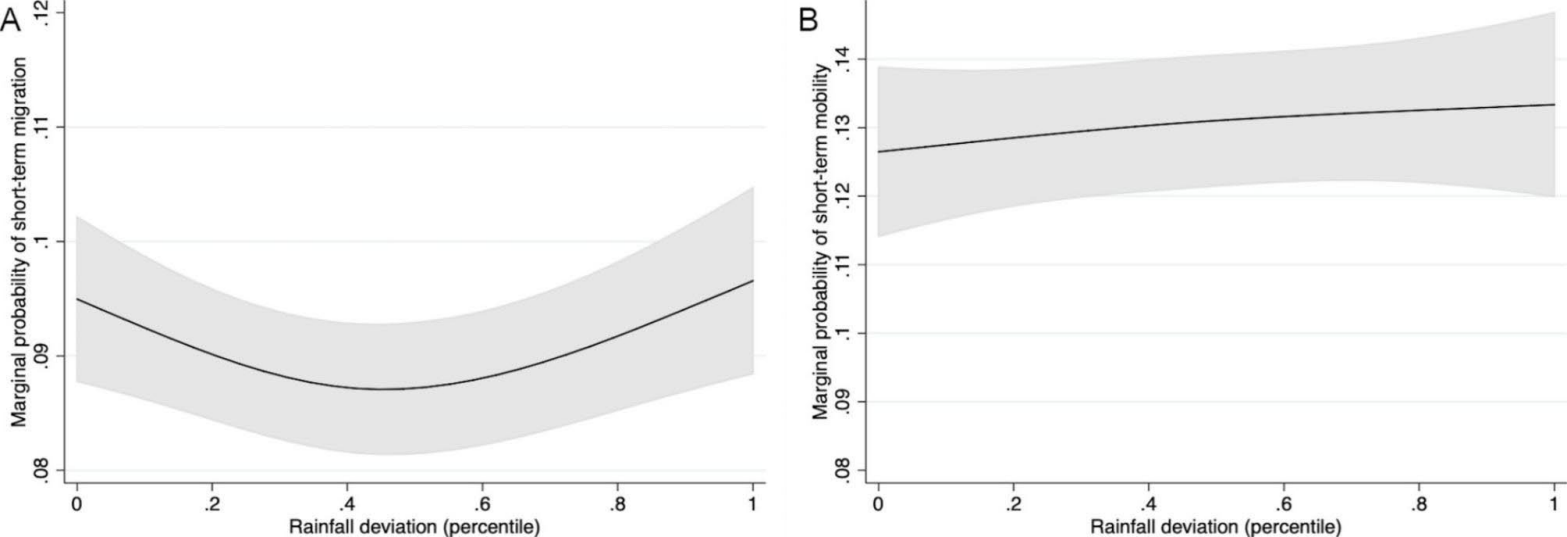
Marginal probabilities of short-term mobility across the range of percentile deviations among (A) women and (B) men

Marginal predicted probabilities for adjusted short-term mobility across rainfall deviations are shown in Fig. 1A and B. These models indicate that the relationship between rainfall deviations and short-term mobility is U-shaped among women, with higher probabilities at low and high deviations (e.g., drought and heavy rain conditions). We did not observe a non-linear association between rainfall deviations and the probability of short-term migration among men, with a relatively flat line suggesting little association between precipitation anomalies and short-term mobility in this group.

Among women, these findings equate to a marginal risk ratio (RR) comparing the 15th rainfall percentile (the cut-off previously defined as drought [24, 25]) to the 50th percentile of 1.05 (95% CI 1.01–1.08) and a marginal RR comparing the 85th percentile to the 50th percentile of 1.06 (95% CI 1.02–1.10). Among men, there was a slight increase in the probability of mobility as rainfall deviations increase. However, we could not rule out a null association among men: this equates to marginal RR comparing the 15th to the 50th percentile of 0.99 (95% CI 0.94–1.03) and the 85th to the 50th percentile of 1.02 (95% CI 0.97–1.07). Marginal RRs by country are presented in Supplemental Fig. 1. In addition to differences between men and women, these findings also indicate between-country variation. Drought was positively associated with higher risk of short-term mobility in 3 countries among women and 2 countries among men; heavy rainfall was associated with higher risk of mobility in 5 countries among women and 4 countries among men. Contrary to pooled findings, we found some instances where rainfall deviations were negatively associated with short-term mobility: drought was negatively associated with mobility in 1 country among women and 3 countries among men, and heavy rainfall was negatively associated with mobility in 1 country among women and 1 country among men.

Table 3 shows marginal risk ratios by marital status, further stratified by gender. Among women, we found evidence for effect modification by marital status (interaction p = 0.0001). These findings indicate that married women demonstrated positive associations between both rainfall deviations (e.g., drought [marginal RR = 1.11, 95% CI 1.07–1.16] and heavy rains [marginal RR = 1.04, 95% CI 1.01–1.07]) and short-term mobility. Among unmarried women, we did not observe an association between drought and short-term mobility (marginal RR = 1.00, 95% CI 0.96–1.03), but we did observe an association between heavy rains and short-term mobility (marginal RR = 1.09, 95% CI 1.05–1.14). Among men, we did not find evidence for effect modification (interaction p = 0.93), nor did we find evidence for any association between rainfall deviations and short term mobility, regardless of marital status.

**Table 3:** Associations between rainfall percentile deviations and short-term mobility stratified by marital status and gender

	Drought		Heavy rainfall	
Gender	Married	Unmarried	Married	Unmarried
**Women**	1.11*** (1.07, 1.16)	1.00 (0.96, 1.03)	1.04* (1.01, 1.07)	1.09*** (1.05, 1.14)
**Men**	1.03 (0.98, 1.08)	1.02 (0.98, 1.07)	0.99 (0.95, 1.04)	0.98 (0.94, 1.03)

Coefficients are presented as marginal risk ratios resulting from logistic regression models with 95% confidence intervals in parentheses. Drought compares the 15th percentile rainfall deviation to the 50th percentile rainfall deviation; heavy rainfall compares the 85th percentile rainfall deviation to the 50th percentile rainfall deviation. All models control for age, wealth quintile, household size, education level, urban (versus rural), and literacy.

Asterisks denote level of significance ***p < 0.001 **p < 0.01 *p < 0.05

## Discussion

Using survey data from 23 countries in sub-Saharan Africa, we found a positive association between precipitation anomalies and short-term mobility among women, but not men. While the predicted increase in probability of mobility among women is relatively small (5–6%), the implications are broad given the growing exposure to precipitation extremes: between 1980 and 2014, 363 million people in sub-Saharan Africa were affected by drought [30], and this figure is expected to increase by 426.6% by 2081–2100 [12]. This finding may have broad health implications, as female migrants face distinct health and social challenges. Mobility may limit women’s access to reproductive health care, and female migrants are particularly vulnerable to sexual abuse and violence [19].

There are several potential reasons for the difference in findings by gender. We do not have information on the reasons behind mobility in this analysis; however, there is increasing evidence that over the past three decades women’s internal migration in sub-Sarahan Africa is increasing not only due to nuptiality, but for labor purposes [31, 32], previously a male phenomenon. Women’s labor migration is less likely to be permanent, as women are less likely to gain employment in the formal sector, particularly in sub-Saharan Africa [33]. The income shocks associated with extreme precipitation events may therefore lead to female short-term labor mobility. We considered only short-term mobility but did not have a measure for long-term or permanent migration; men may be more likely to seek work elsewhere and therefore migrate for longer periods of time. Furthermore, country-level analyses revealed settings where rainfall deviations were positively associated with short-term mobility among men. Indeed, several studies have shown that drought may increase men’s economic migration, which may require longer-term stays away from home [7, 34, 35]. These men would not be captured in DHS surveys. Our findings are also consistent with a study in Mali that found that women and children (but not men) migrated in the short-term after drought [5]. In the Malian setting, women departed with children to stay with relatives to stabilize food supply in the household during drought-induced shortages. More work is needed to elucidate the mechanisms behind gender differences in precipitation-related short-term mobility.

Our findings also point to differences between marital status among women. We found that, among married women, there were positive associations between both rainfall deviation extremes (drought and heavy rains) and mobility; however, among unmarried women, there was only a positive association for heavy rains and not for drought. One potential explanation for this is that unmarried women may be more impacted by drought-induced income shocks as they do not have additional spousal support, and therefore may have less financial ability to fund a move. This is supported by some of the literature, which suggests that the poor and most vulnerable to climate shocks may in fact be the least likely to migrate due to financial and structural constraints [36, 37]. Alternatively, unmarried women may face less pressure to make up for lost earnings during drought-induced income shocks, as they are less likely to have children to support.

We found between-country differences in the relationships between rainfall deviations and short-term mobility. This is unsurprising, given the important differences across countries, including economies, infrastructure, and employment opportunities, that may shape individuals’ responses to climate shocks. Counter to our pooled findings, we identified instances where rainfall deviations were negatively associated with short-term mobility among both women and men. This could be driven by reductions in income resulting from climate shocks, as funds are required for migration.

The major limitation of this study is its outcome measure. While the DHS asks about short-term mobility, it does not follow up with questions regarding the reasons for migrating, nor are respondents asked their destination. In addition, we had only one timepoint and therefore did not capture longer term migrants. Future work, particularly using longitudinal data documenting both in- and out-migration, should attempt to classify mobility patterns following climate events by destination and time spent as a migrant, and should incorporate reason for migrating. Finally, we do not assess potential down-stream impacts of drought-induced migration, including health impacts. This is an additional line of inquiry that should be addressed in the future.

## Conclusion

Precipitation anomalies are expected to increase in intensity and frequency over the coming decades as a result of climate change. Given this, more research is imperative to better understand environmental mobility in order to best tailor interventions aimed at mitigating potential health consequences. Moreover, further studies are needed to better understand the gendered differences in terms of health outcomes for men and women in order to aid appropriate culturally-tailed interventions.

## Electronic supplementary material

Below is the link to the electronic supplementary material.

Supplementary Material 1

## Data Availability

All DHS data are publicly available from https://dhsprogram.com/data/. CHIRPS precipitation data are publicly available from https://www.chc.ucsb.edu/data/chirps.

## List of Abbreviations

DHS: Demographic and Health Surveys
EA: Enumeration area
RR: Risk ratio
