# Supplementary material for: Do precipitation anomalies influence short-term mobility in sub-saharan Africa? An observational study from 23 countries
Source: BMC Public Health. 2023 Feb 22;23:377. doi: 10.1186/s12889-023-15264-z (PMC9948323; doi:10.1186/s12889-023-15264-z)
Supplement: Supplementary file 1 — Supplementary Material 1 [file 12889_2023_15264_MOESM1_ESM.pdf]

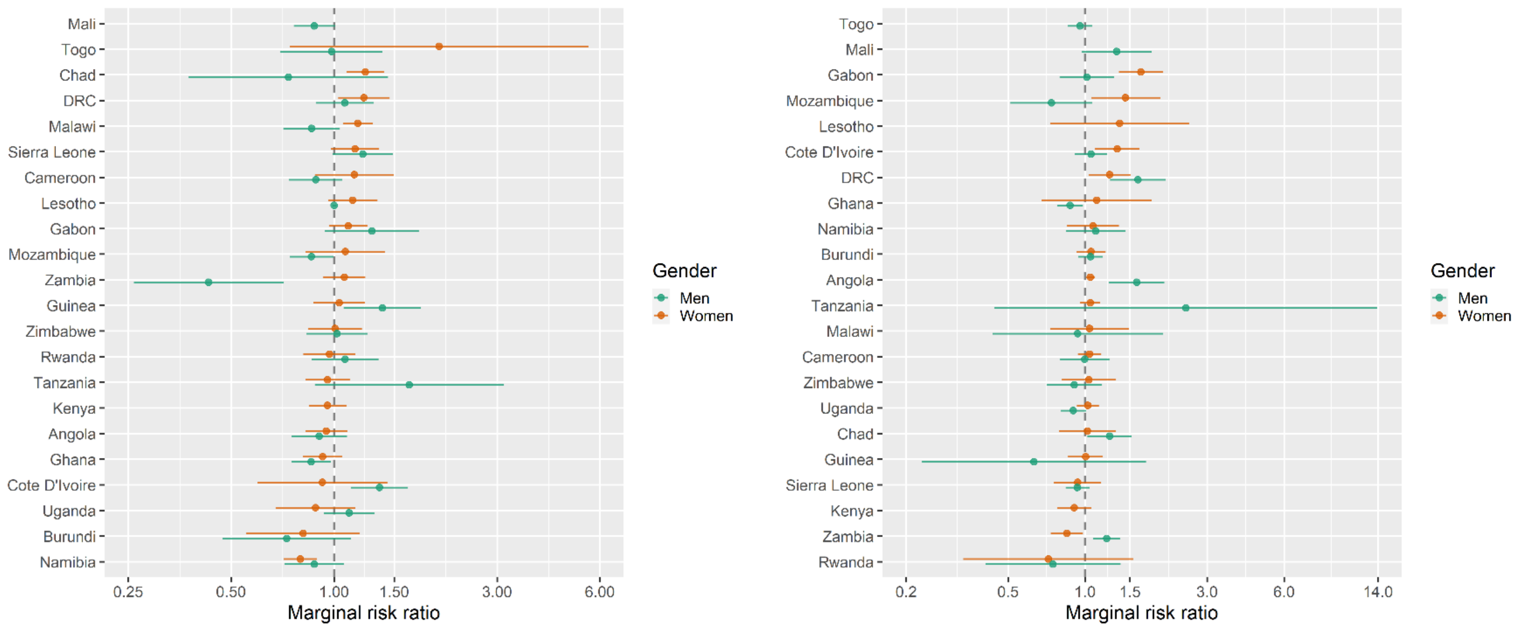

Supplemental Figure 1. Country-level associations between drought (left) and heavy rainfall (right) and short-term mobility, by gender. Drought compares the 15th percentile rainfall deviation to the 50th percentile rainfall deviation; heavy rainfall compares the 85th percentile rainfall deviation to the 50th percentile rainfall deviation. Results not shown for countries with insufficient variation in outcome data.
